# Supplementary figures and images for: Gene co-expression network analysis reveal core responsive genes in Parascaris univalens tissues following ivermectin exposure
Source: PLoS One. 2024 Feb 15;19(2):e0298039. doi: 10.1371/journal.pone.0298039 (PMC10868809; doi:10.1371/journal.pone.0298039)

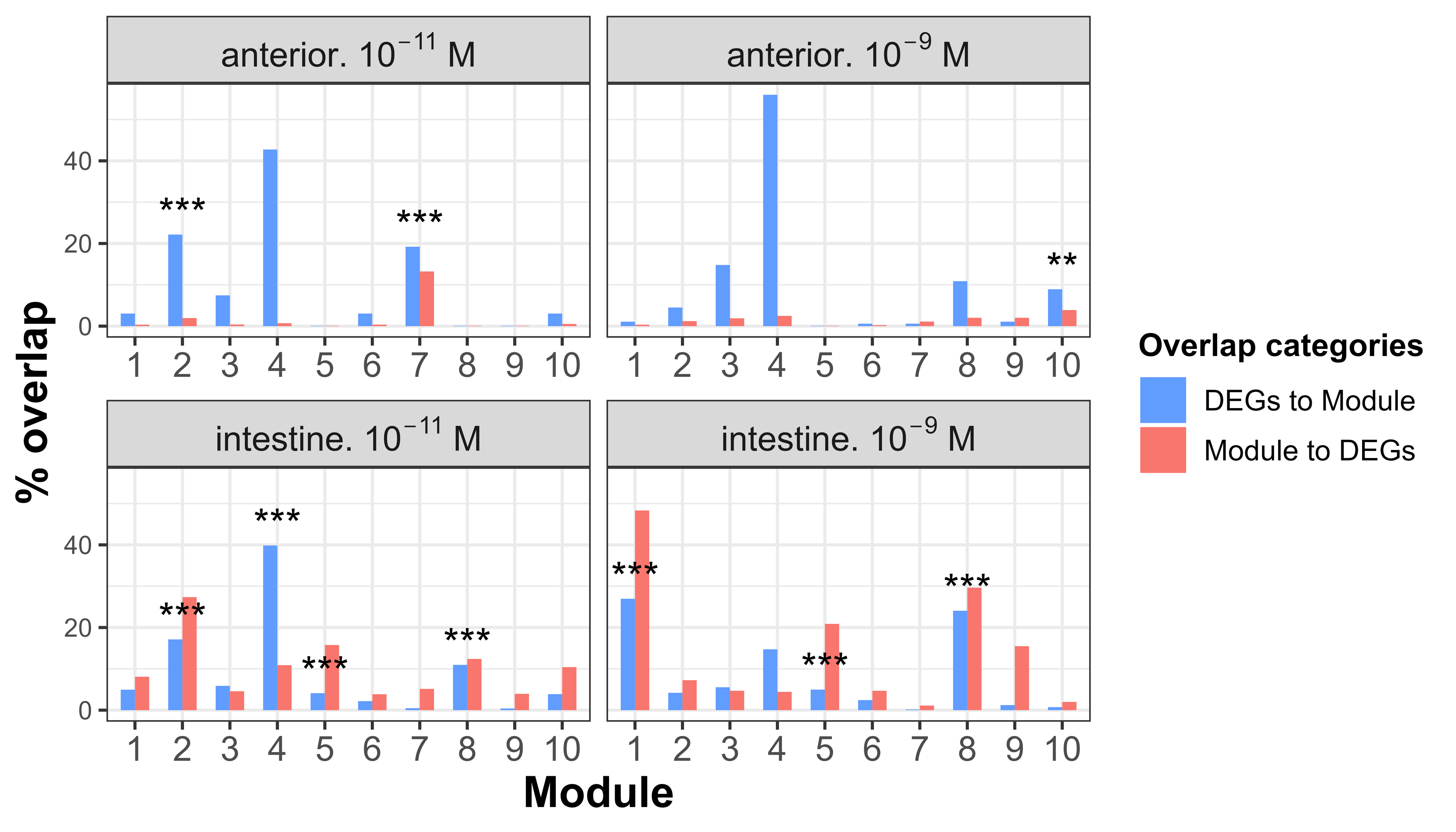

Supplement: S1 Fig — The bar charts represent the percentage overlap between differentially expressed genes (DEGs) and gene modules, illustrated for both 10−9 M and 10−11 M concentrations. Blue bars indicate the percentage of DEGs within each module, while red bars show the percentage of each module’s genes that are differentially expressed. Statistical significance is marked by asterisks above the bars (***p<0.001, **p<0.01). (TIF) [file pone.0298039.s017.tif]

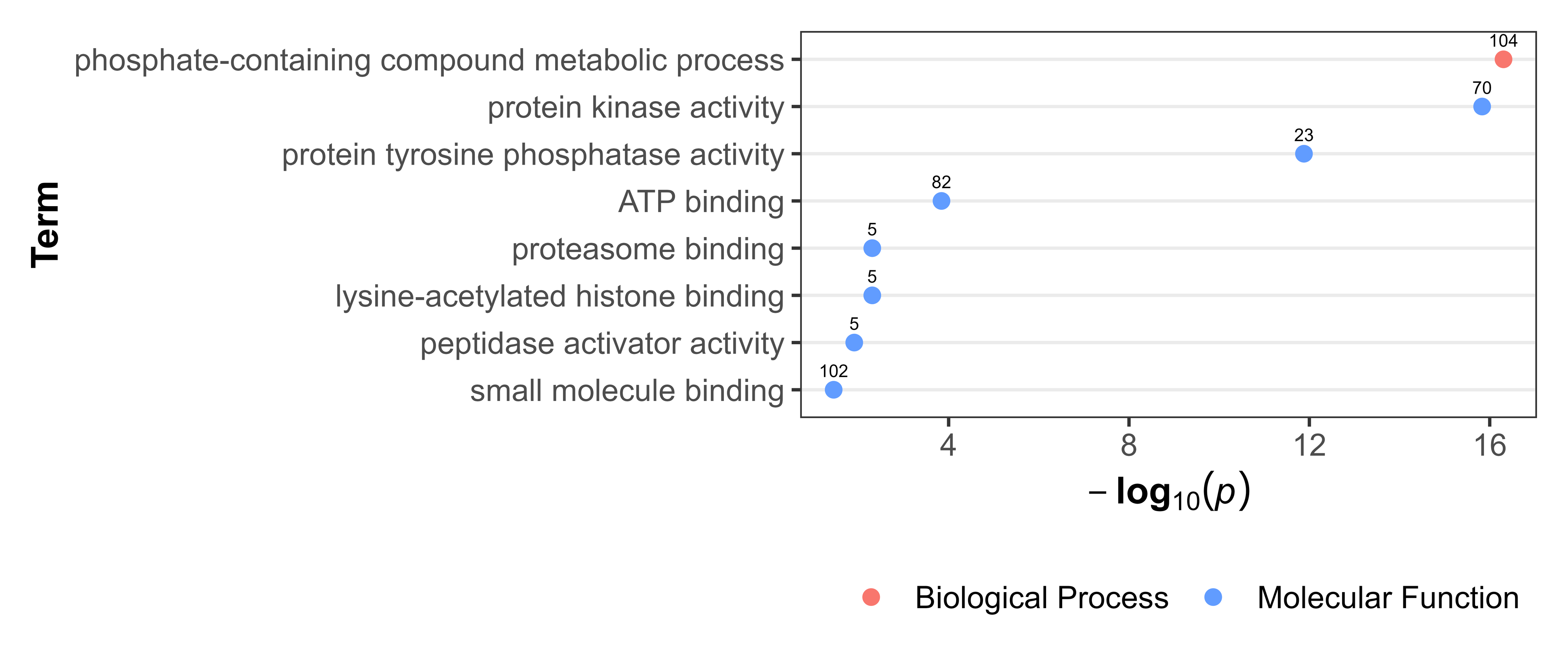

Supplement: S2 Fig — The data points, color-coded by ontology classification-Biological Process (red), Cellular Component (green), and Molecular Function (blue) are plotted against their respective -log10 transformed p-values, emphasizing the significance of each term. The numerical labels adjacent to each data point indicate the count of genes. (TIF) [file pone.0298039.s018.tif]

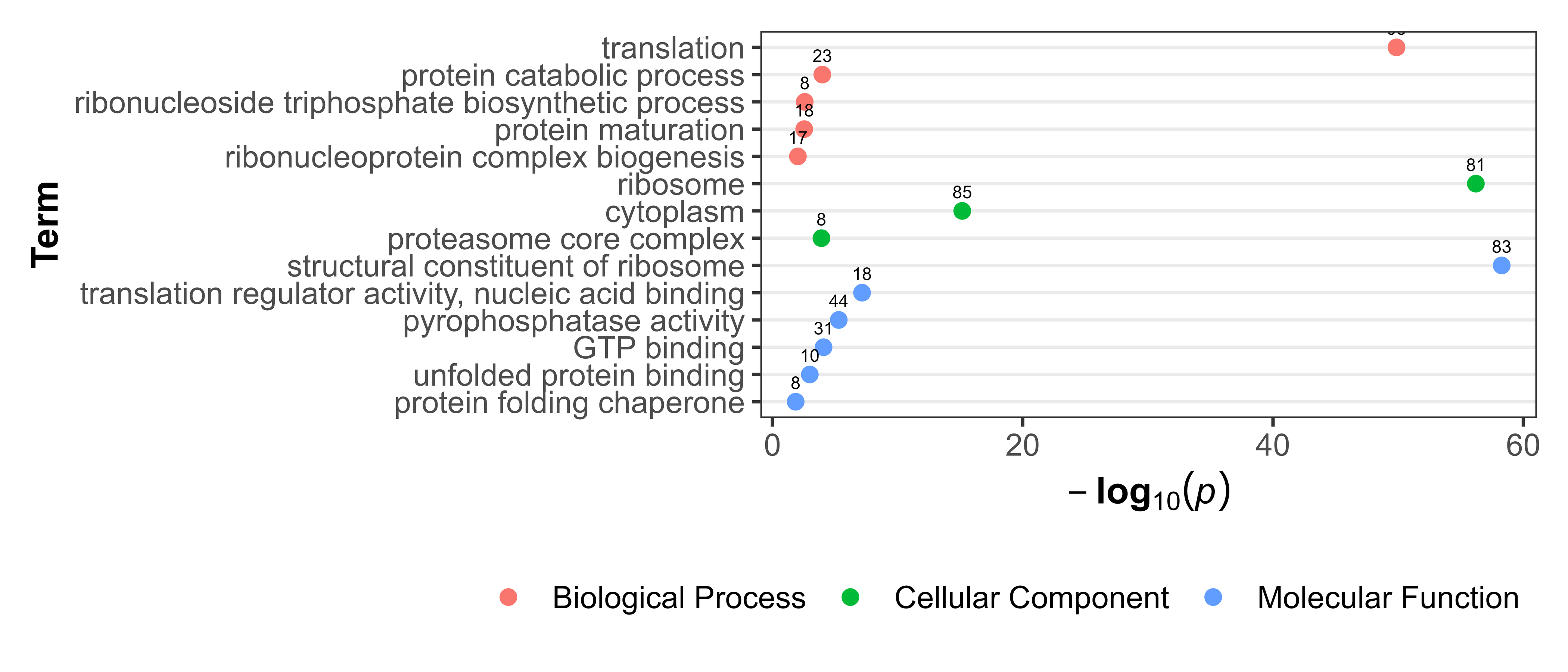

Supplement: S3 Fig — The data points, color-coded by ontology classification-Biological Process (red), Cellular Component (green), and Molecular Function (blue) are plotted against their respective -log10 transformed p-values, emphasizing the significance of each term. The numerical labels adjacent to each data point indicate the count of genes. (TIF) [file pone.0298039.s019.tif]

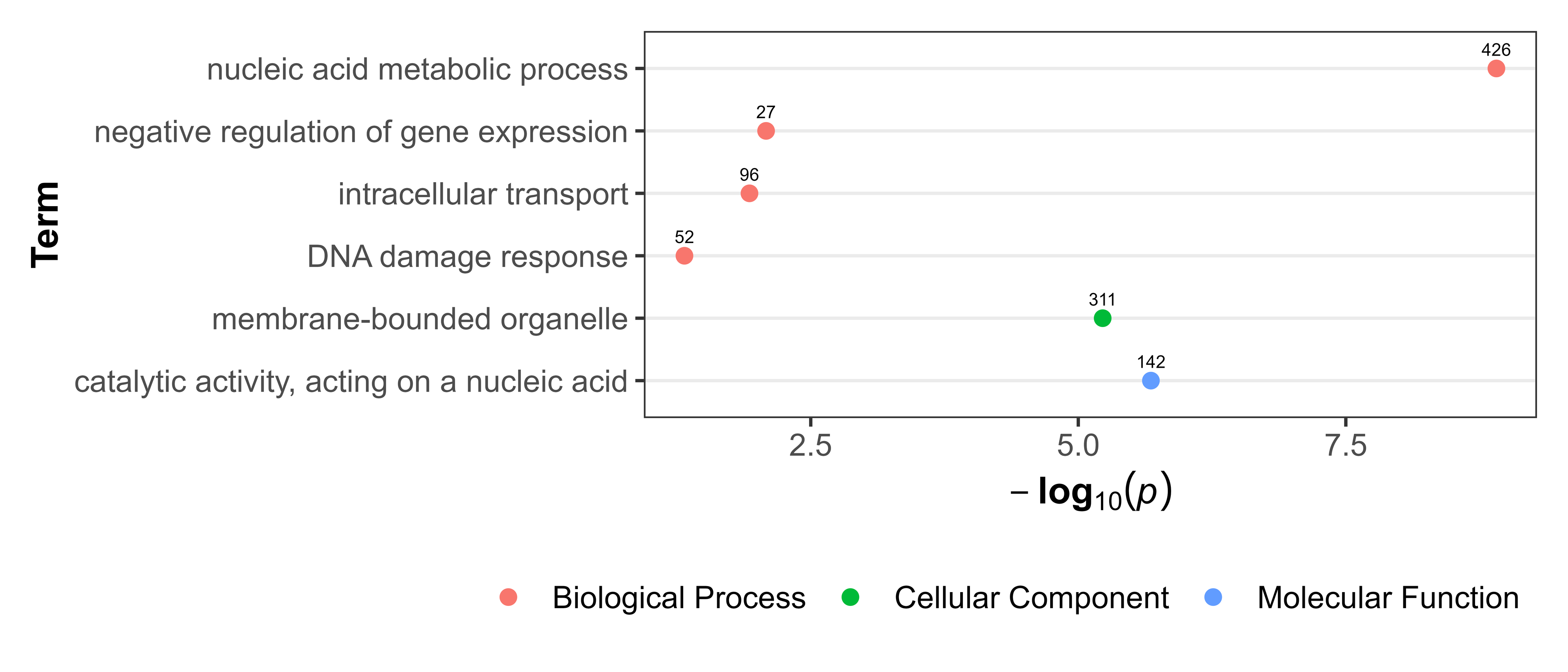

Supplement: S4 Fig — The data points, color-coded by ontology classification-Biological Process (red), Cellular Component (green), and Molecular Function (blue) are plotted against their respective -log10 transformed p-values, emphasizing the significance of each term. The numerical labels adjacent to each data point indicate the count of genes. (TIF) [file pone.0298039.s020.tif]

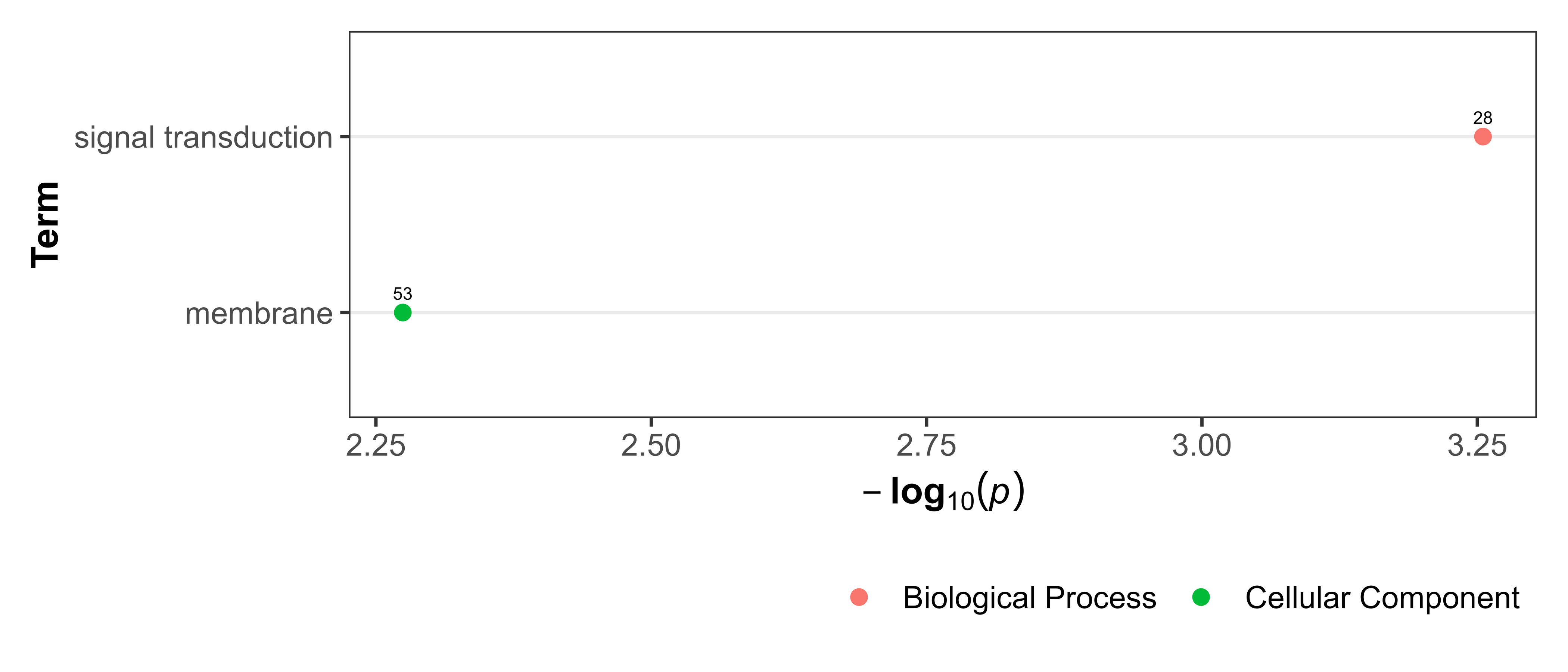

Supplement: S5 Fig — The data points, color-coded by ontology classification-Biological Process (red), Cellular Component (green), and Molecular Function (blue) are plotted against their respective -log10 transformed p-values, emphasizing the significance of each term. The numerical labels adjacent to each data point indicate the count of genes. (TIF) [file pone.0298039.s021.tif]

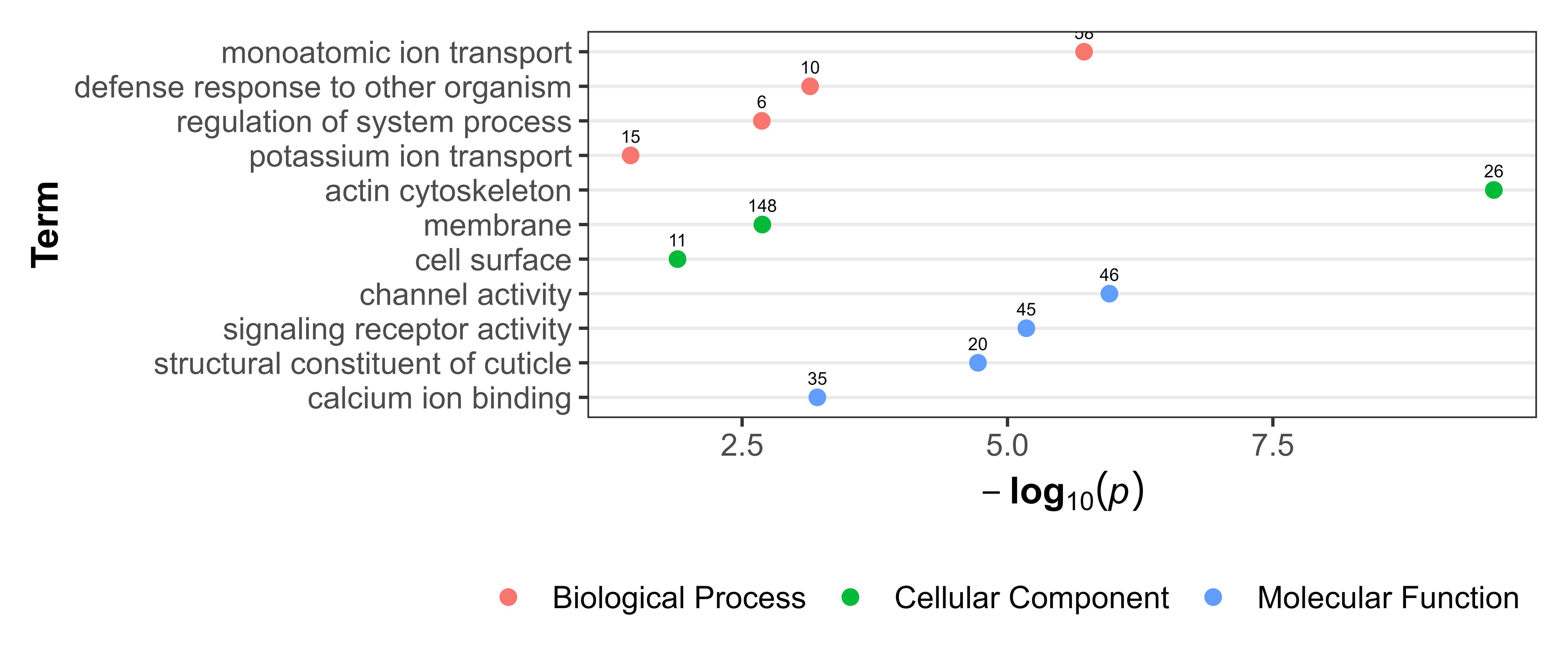

Supplement: S6 Fig — The data points, color-coded by ontology classification-Biological Process (red), Cellular Component (green), and Molecular Function (blue) are plotted against their respective -log10 transformed p-values, emphasizing the significance of each term. The numerical labels adjacent to each data point indicate the count of genes. (TIF) [file pone.0298039.s022.tif]

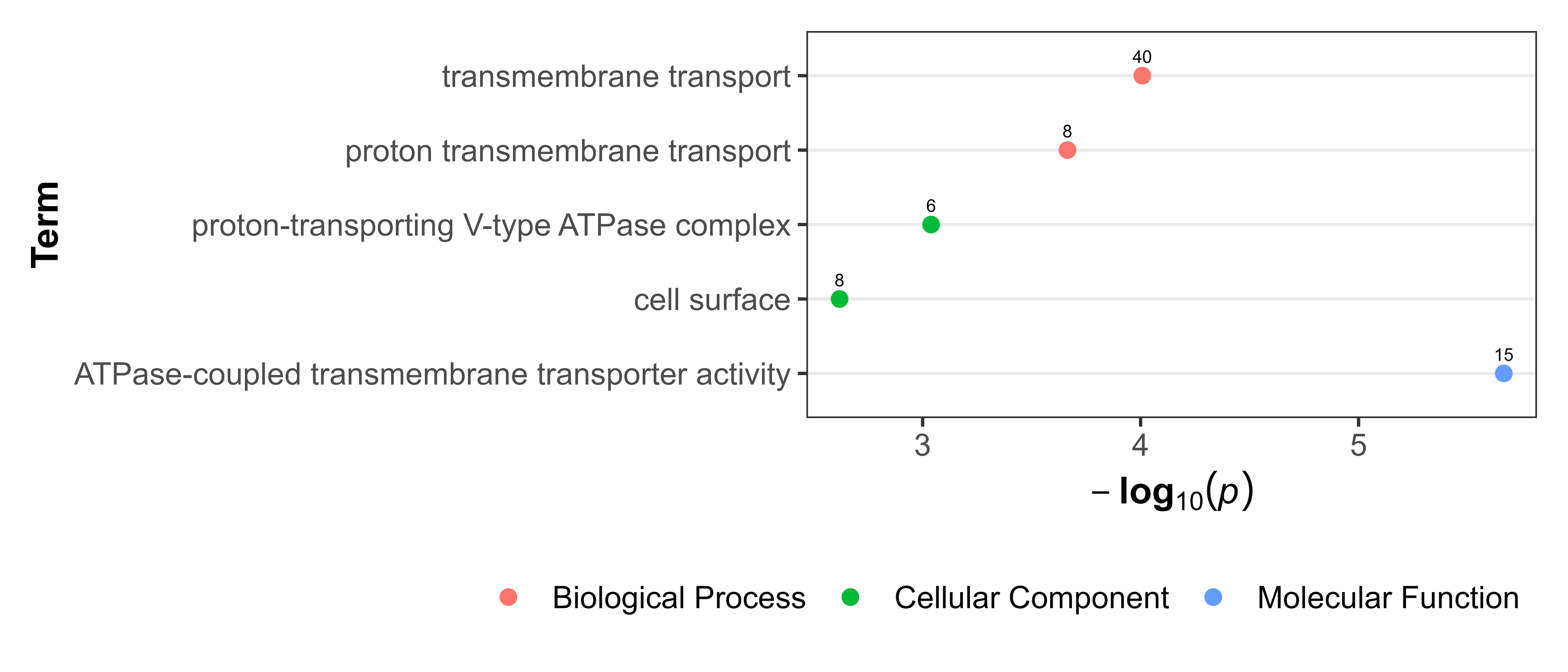

Supplement: S7 Fig — The data points, color-coded by ontology classification-Biological Process (red), Cellular Component (green), and Molecular Function (blue) are plotted against their respective -log10 transformed p-values, emphasizing the significance of each term. The numerical labels adjacent to each data point indicate the count of genes. (TIF) [file pone.0298039.s023.tif]

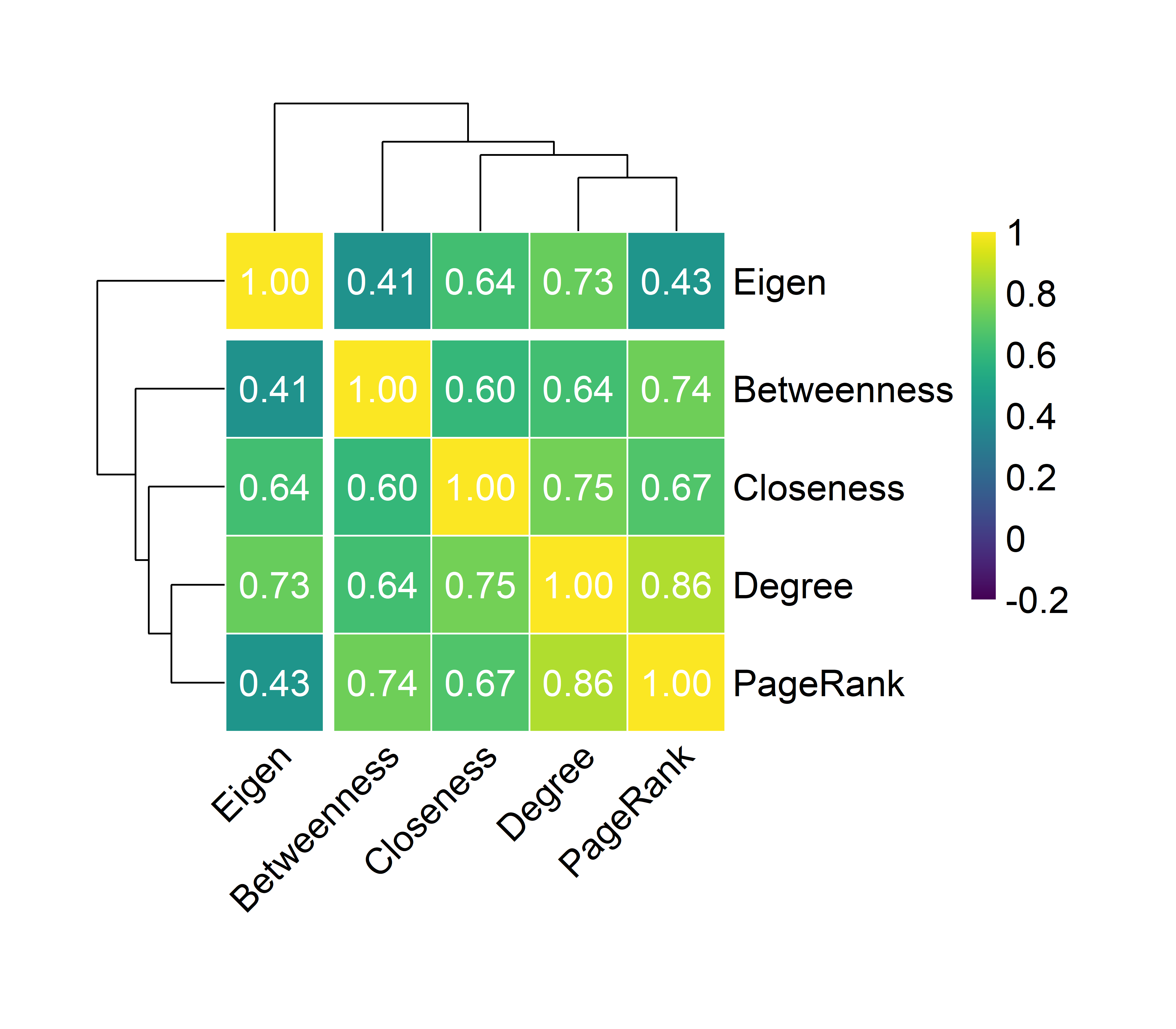

Supplement: S8 Fig — The color-coded matrix displays the Spearman correlation coefficients between different centrality measures, including Eigenvalue, Betweenness, Closeness, Degree, and PageRank, calculated for nodes within the consensus network. Values close to 1 indicate a high positive correlation, illustrated by a gradient from yellow to green, whereas values close to 0 imply no correlation, depicted in purple. The dendrogram reflects the hierarchical clustering based on the correlation values, grouping similar centrality measures. (TIF) [file pone.0298039.s024.tif]
